# Molecular Decoration and Unconventional Double Bond Migration in Irumamycin Biosynthesis

**DOI:** 10.3390/antibiotics13121167

**Published:** 2024-12-03

**Authors:** Vera A. Alferova, Anna A. Baranova, Olga A. Belozerova, Evgeny L. Gulyak, Andrey A. Mikhaylov, Yaroslav V. Solovev, Mikhail Y. Zhitlov, Arseniy A. Sinichich, Anton P. Tyurin, Ekaterina A. Trusova, Alexey V. Beletsky, Andrey V. Mardanov, Nikolai V. Ravin, Olda A. Lapchinskaya, Vladimir A. Korshun, Alexander G. Gabibov, Stanislav S. Terekhov

**Affiliations:** 1 Shemyakin-Ovchinnikov Institute of Bioorganic Chemistry, Miklukho-Maklaya 16/10, Moscow 117997, Russia; anjabaranowa@list.ru (A.A.B.); o.belozyorova@gmail.com (O.A.B.); mikhaylov_andrey@yahoo.com (A.A.M.); yaroslavsolovev78@gmail.com (Y.V.S.); droplbox38@gmail.com (M.Y.Z.); asinichich@yandex.ru (A.A.S.); anton2rin@gmail.com (A.P.T.); katatrusova532@gmail.com (E.A.T.); v-korshun@yandex.ru (V.A.K.); gabibov@gmail.com (A.G.G.); 2 Department of Chemistry, Lomonosov Moscow State University, Leninskie Gory 1, Moscow 119991, Russia; 3 Institute of Bioengineering, Research Center of Biotechnology of the Russian Academy of Sciences, Leninsky Prospect 33-2, Moscow 119071, Russia; mortu@yandex.ru (A.V.B.); mardanov@biengi.ac.ru (A.V.M.);; 4 Gause Institute of New Antibiotics, B. Pirogovskaya, 11, Moscow 119021, Russia; lapchinskaya.olda@mail.ru

**Keywords:** type I polyketide synthase, olefin shift, biosynthetic gene cluster, epoxidation, antibiotic, *Streptomyces* sp.

## Abstract

Irumamycin (Iru) is a complex polyketide with pronounced antifungal activity produced by a type I polyketide (PKS) synthase. Iru features a unique hemiketal ring and an epoxide group, making its biosynthesis and the structural diversity of related compounds particularly intriguing. In this study, we performed a detailed analysis of the *iru* biosynthetic gene cluster (BGC) to uncover the mechanisms underlying Iru formation. We examined the *iru* PKS, including the domain architecture of individual modules and the overall spatial structure of the PKS, and uncovered discrepancies in substrate specificity and iterative chain elongation. Two potential pathways for the formation of the hemiketal ring, involving either an olefin shift or electrocyclization, were proposed and assessed using ^18^O-labeling experiments and reaction activation energy calculations. Based on our findings, the hemiketal ring is likely formed by PKS-assisted double bond migration and TE domain-mediated cyclization. Furthermore, putative tailoring enzymes mediating epoxide formation specific to Iru were identified. The revealed Iru biosynthetic machinery provides insight into the complex enzymatic processes involved in Iru production, including macrocycle sculpting and decoration. These mechanistic details open new avenues for a targeted architecture of novel macrolide analogs through synthetic biology and biosynthetic engineering.

## 1. Introduction

The realm of natural macrolides has provided mankind with clinically important drugs: antibacterial erythromycins, immunosuppressive tacrolimus, antifungal amphotericins, anti-parasitic avermectins, anticancer/immunosuppressive/rejuvenating agent rapamycin, to name a few. The bizarre diversity of macrolactone core structures, the endless opportunities for shuffling hydrophobic (alkane, polyene) and hydrophilic (hydroxyls, carboxyls, etc.) functions, together with the possibilities for macrolide–peptide hybrids and for further glycosylation make these compounds an inexhaustible source of potential drugs. Polyketide synthases (PKSs) are unique multifunctional enzymes responsible for macrolide core assembly [1,2]. PKSs have a modular organization: the polyketide backbone is extended in a linear fashion by individual modules, followed by macrolactone ring closure. The modular properties make PKSs attractive as targets for the rational engineering of novel chemical production [3,4]. The biosynthesis of important macrolide-based drugs and, in particular, their PKSs have been well studied [5,6,7,8]. In general, research on PKS architecture is essential for evaluating, accessing, and expanding the diversity of polyketide natural products [9].

**Figure 1:**
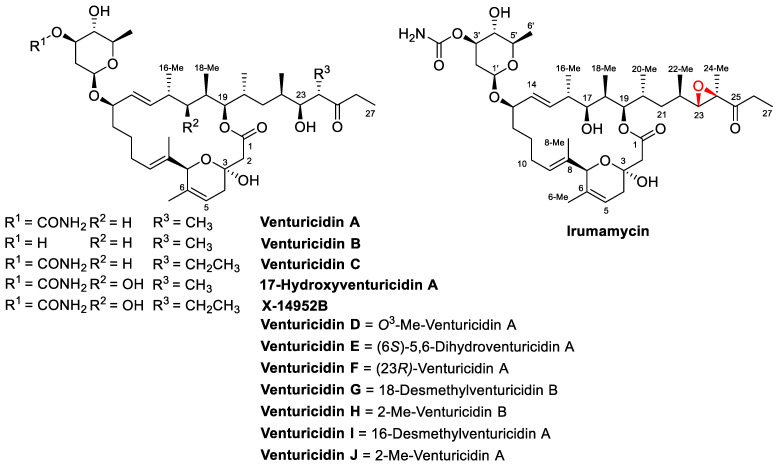
Structures of some venturicidin-type compounds.

Many 20-Membered macrolides have been found in various marine and soil microorganisms, e.g., miyakolide [10], amphidinolide U [11], palmerolide [12], iriomoteolide-1a [13], ammocidins [14], levantilides [15,16,17], rickiols [18], formicolides [19], gibbosolide A [20], shuangdaolides [21], krasilnikolides [22], etc. However, the most studied 20-membered macrolides are compounds of the venturicidin family: venturicidins/X-14952B/irumamycin (Figure 1). The first venturicidin compound was discovered more than sixty years ago and its potential agricultural use was immediately claimed [23]. Later studies [24,25,26,27,28,29] yielded structures and stereochemistry of venturicidin-type compounds (Figure 1), but the potential of their bioactivity was not developed further. Recently, compounds of the venturicidin family have regained attention as F_0_·F_1_-ATPase/synthase inhibitors [30,31,32], potential antibiotic adjuvants [33,34], and fungicides for controlling *Fusarium* [35]. Very recently, venturicidin A was recognized as a useful antifungal agent for controlling gray mold disease caused by *B. cinerea* on tomato and blueberry fruits. The antibiotic disrupted cell membrane integrity and induced intracellular ROS accumulation, leading to the downregulated expression of pathogenicity-related genes, as well as reduced pathogen penetration on onion epidermis [36]. In contrast, the biological activity of irumamycin is much less studied. Irumamycin was once reported to have antiparasitic activity superior to that of venturicidin [37]. The main structural feature of irumamycin compared to venturicidins is the 23,24-epoxy group (Figure 1, highlighted in red). Epoxides are found in some antibiotics, e.g., fosfomycin, and are reactive with amines and may therefore contribute to irreversible enzyme inactivation.

Venturicidin congener biosynthesis was reported only recently [38,39,40]. Here, we report the biosynthesis of irumamycin (Iru), a venturicidin-related compound produced by *Streptomyces* sp. INA-Ac-5812 [22], along with the antibacterial lipoglycopeptides gausemycins, which exhibit potent Ca^2+^-dependent activity against Gram-positive pathogens and are completely devoid of antifungal activity [41,42].

## 2. Results and Discussion

**Figure 2:**
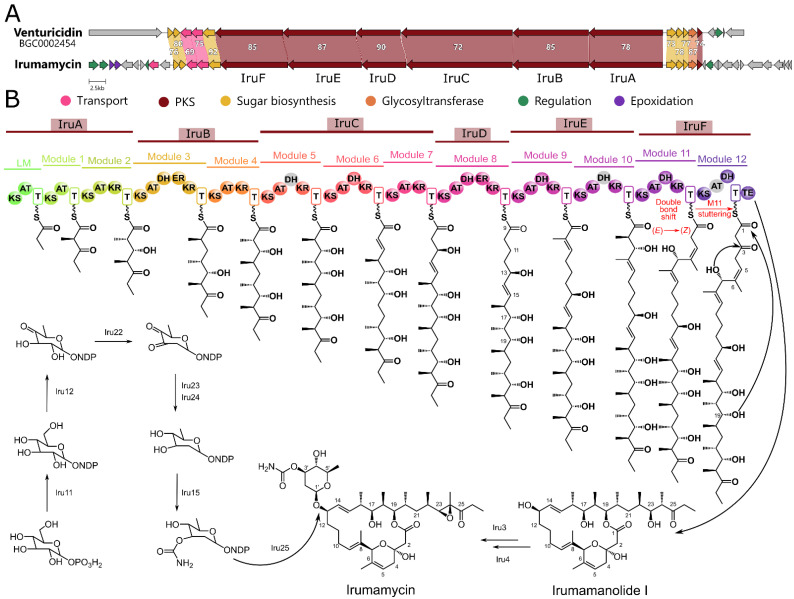
Proposed scheme of Iru biosynthesis. (**A**) Comparison of *ven* [38] and *iru* (this work) BGCs. Homologous proteins are marked with colors with similarity (%) indicated on the labels. (**B**) The modular organization (module domains are highlighted with the same color code) of the *iru* PKS with the proposed scheme of backbone biosynthesis and tailoring steps.

Analysis of the complete genome sequence of *Streptomyces kanamyceticus* INA-Ac-5812 revealed a large BGC, presumably involved in the biosynthesis of Iru (Figure 2). The *iru* BGC shows a high degree of similarity to the *ven* BGC [38] (Figure 2A,B, Table S1).

The key difference between Iru and other related polyketides is the epoxidation at the C23–C24 position. According to early studies on the mechanism of Iru biosynthesis, the sequential accumulation of intermediate biosynthesis products, irumanolide I and irumanolide II, was observed in the culture broth of the producer strain [43]. These compounds are Iru precursors, lacking the carbohydrate fragment and the epoxide in the side chain (Figure S4). This observation is consistent with the absence of a DH domain in the M2 module, indicating that the synthesized polyketide contains a hydroxyl group at position 23, which subsequently undergoes dehydration and epoxidation by tailoring enzymes.

Additionally, a related compound, X-14952B (Figure 1), which contains an ethyl group at position 24 instead of a methyl group, was also detected in the culture fluid of the producer strain. X-14952B does not undergo dehydration and retains the hydroxyl group at position 23, suggesting an enzyme specificity dependent on the substituent at position 24. The most obvious candidate for epoxidation is Orf4, which encodes an F420/flavin-dependent oxidoreductase (Figure S6). Its closest structural homolog is a luciferase-like monooxygenase from Bacillus cereus (PDB ID: 2B81), whose enzymatic activity and substrate specificity have not yet been described. Orf3 encodes a protein of unknown function (DUF5995) that may be involved in Orf4 functionality (Figure S7). However, it has no structural homolog that has been functionally described. Notably, these enzymes are absent in BGCs that mediate the production of venturicidin [38] and X-14952B [40], which lack the epoxide fragment. However, further studies are required to determine their exact role and substrate specificity.

In the final stage of Iru biosynthesis, the precursor synthesized by the PKS is glycosylated. The biosynthesis of the sugar moiety is mediated by a set of enzymes similar to those involved in the biosynthesis of venturicidin [38] and concanamycin [44]. Following glycoside synthesis, glycosylation of the previously synthesized polyketide is catalyzed by the enzyme Iru25, which is homologous to several glycosyltransferases described in various streptomycetes (Figure 1).

To support the proposed biosynthetic scheme, we conducted a detailed analysis of the PKS domains. The domain architecture of the *iru* BGC includes 12 modules. As expected, the alignment of the ketosynthase domains (KS) revealed a conserved glutamine residue in the active site of the loading module KS (KSQ) instead of the cysteine found in downstream domains (Figure S1).

Alignment of the amino acid sequences of the acyltransferase domains revealed some discrepancies between the predicted and observed specificities in the isolated molecule. The AT domain of the eighth module (M8 AT) has conserved motifs characteristic of methylmalonyl-specific domains (Figure S2), yet the structures of irumamycin and all related macrolides lack a methyl group at this position.

Eight methylmalonyl-specific AT domains (LM, M1-M5, M9, M10) contain typical sequences xVDVxQ, GHSQGE, xxSH for motifs I, II, and III, respectively, which are associated with methylmalonate specificity [45,46]. According to the structure of the synthesized macrolides, the remaining domains should have malonate specificity. Indeed, the domains in modules M6, M7, M8, and M11 contain sequences characteristic of malonate-specific domains [45,46] xTxYTQ, GHSI(V)GE, xAFN for motifs I, II, and III, respectively. However, the structures of the isolated macrolides and previous studies with isotopically labeled precursors [47] showed complete agreement with the substrate specificity of the domains proposed in Figure 1, including the unambiguous insertion of a malonate, rather than a methylmalonate residue, at position C9-C10 by the M8 module. Taking into account the same motifs in the M8 AT module in the *ven* BGC [38] and the lack of mention of C10-Me congeners in the literature, the selectivity of the M8 module is provided by the ketosynthase domain, rather than the acyltransferase domain. A similar situation was previously observed for the large polyol macrolides PM100117/8 [48] and caniferolides [49].

The structure of the AT domain of the last module M12 shows that this domain not only has low amino acid similarity to all conserved motifs, but also lacks serine in the catalytic center (Figure S2). In motif II, serine GHS is replaced by glycine, suggesting that this domain is inactive, since this mutation results in a complete loss of AT domain activity [50]. A possible explanation for this fact is iterative chain elongation by the previous domain M11. Iterative repetition of the domain, so-called “stuttering”, has previously been described to explain the biosynthesis of polyketides two atoms longer than expected from the PKS architecture [51,52]. A model including both the skipping and iterative “stuttering” of the domain has been proposed to explain the unusual biosynthesis of epothilone [53].

**Figure 3:**
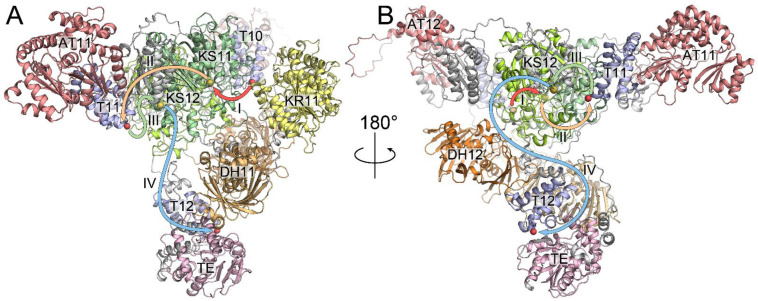
Front (**A**) and back (**B**) sides of the IruF/IruE complex. PKS domains in the AlphaFold3 model are colored green (KS), red (AT), yellow (KR), orange (DH), blue (T), and pink (TE). Domain numeric attributes indicate PKS modules. Arrows indicate the proposed pathway of the growing chain. Roman numerals indicate chain transfer steps.

To uncover the potential mechanism of the repetitive addition of malonyl moieties during the final steps of Iru biosynthesis, we computed an AlphaFold3-based structure of the complex of IruF with the C-terminal fragment of IruE, including CP and the docking domain (Figure 3 and Figure S5). The resulting model reveals the close proximity of T10-KS11 and AT11-T11-KS12 fragments. On the contrary, AT12, which is inactive and methylmalonyl-specific, is far from both T12 and T11. Hence, we believe that it plays only a structural role and does not participate in chain elongation. According to the generated structural model, we suggest that the growing chain attached to the T10 is subjected to carbonyl reduction and dehydration with a double bond shift followed by its loading onto KS11 (I). The malonyl-specific AT11 loads malonyl onto T11, and the growing chain is transferred by KS11 to T11 (II) with malonyl fragment elongation. We speculate that the KS12 domain’s specificity is controlled by the DH12 domain, which may mediate hemiketal formation. Hence, the growing chain is transferred from T11 to KS12 (III) after the final modification. The T12 domain is very far from the inactive AT12. However, T12 has extended flexible loops that allow it to reach AT11. Finally, the growing chain is transferred by KS12 to T12 with malonyl fragment elongation (IV), and the TE domain catalyzes macrocyclization.

Alignment of the ketoreductase domains of Iru PKS revealed the presence of the characteristic LDD motif in the domains of modules M2-M4 and M11, which unambiguously classifies them as type B ketoreductase domains. Modules M6, M8, and M9 contain less common amino acid sequences of this motif, IDD and VDD, which have also been identified in other type B ketoreductase domains (Figure S3) [54].

**Figure 4:**
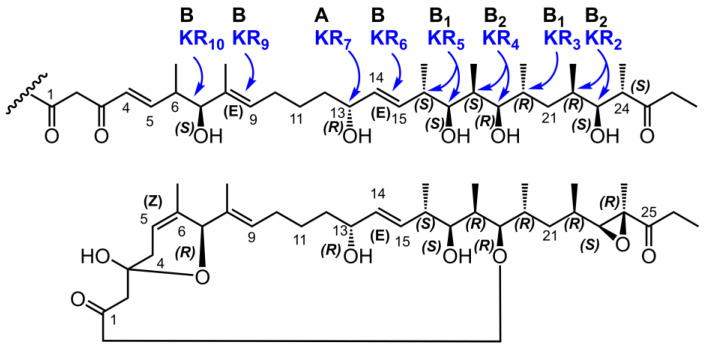
Comparison of the absolute configurations in the Iru backbone, derived from the KR domain types (blue) and previously established by NMR [28].

Additionally, the presence of the YxP motif in the KR domains of M2 and M4 allowed them to be classified as B2-type ketoreductases, while all other B-type ketoreductases contain the YxA domain, which is typical of B1-type ketoreductases. The M7 domain contains a type A ketoreductase, which is confirmed by the absence of a conservative LDD motif and the presence of the WxxxxQ motif (Figure S3) [55]. The absolute configuration predicted based on the types of ketoreductase domains is completely consistent with that previously established based on NMR spectroscopy data [28] (Figure 4).

The structure of Iru contains a double bond in an unusual position. Typically, double bond formation in polyketide biosynthesis involves the formation of an α,β-unsaturated compound after elongation in the module containing the dehydratase (DH) domain. In most cases, a trans-double bond is formed during the dehydration of hydroxyl groups reduced by the B-type ketoreductase domain, while the formation of *cis*-double bonds is usually associated with dehydration of the reduction products by the A-type ketoreductase or with additional isomerization of the primary dehydration product [56,57]. In the *iru* PKS, the formation of a *trans*-double bond in the M6 module is fully consistent with these principles. However, the structure of Iru and its analogs also features a cis-double bond at the C4-C5 position, i.e., a β,γ-double bond.

**Figure 5:**
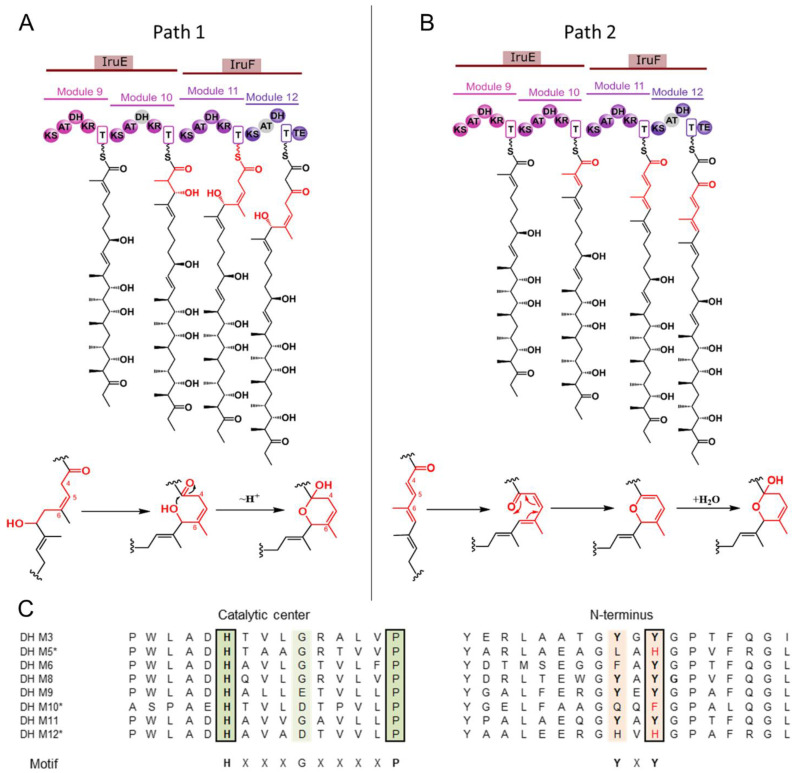
Plausible mechanisms of hemiketal ring formation. Schematic representation of path 1 (**A**) cyclization through PKS-assisted double-bond shift and path 2 (**B**) through cycloaddition. The fragment of Iru backbone involved in hemiketal ring formation is highlighted with red. (**C**) Fragment of *iru* PKS DH domain alignment, presumably inactive domains are marked with asterisks. Key conserved motifs are highlighted with color.

We propose two possible pathways for the formation of this fragment (Figure 5). The first mechanism involves an olefin shift during polyketide synthesis (Path 1, Figure 5A). In this case, the DH domain in module M10 must be inactive to preserve the hydroxyl group for subsequent hemiketal ring formation. Alignment of the DH domains in the *iru* PKS (Figure 5C) reveals the presence of a conserved motif of the catalytic centers HxxxxxxxxP in all domains, including the DH domain of the M11 module, one of which presumably carries an isomerase function. According to the structure of Iru, the DH domains of modules M5 and M12 are predicted to be inactive. The catalytic center of these domains contains a conserved histidine, but they lack a tyrosine in the YxY motif. For DH M10, the same situation is observed, suggesting it might also be inactive.

The shift of the double bond in polyketides from the classical α,β position to the β,γ position was first described and characterized in trans-AT PKS. These polyketide synthases frequently exhibit unusual combinatorial logic: they fuse with nonribosomal peptide synthetases and contain rare domains in their modules, such as methyltransferases, enoyl-CoA hydratases, β-branching domains, and others that are practically not found in cis-AT PKS [58,59]. In trans-AT PDS, the shift of the double bond is mediated by the so-called enoyl isomerase domain (EI domain), which is structurally similar to the DH domain [60]. The isomerization of the double bond by these domains has been well studied for the polyketide bacillaene [60,61] and has also been reported for rhizoxin [62] and corallopyronin [63]. The known EI domains have an amino acid sequence very similar to that of DH domains, with several conservative substitutions. In DH domains, the catalytic center typically features a histidine in the HxxxxxxxxP motif, whereas in most EI domains, the proline is replaced by a hydrophobic amino acid, often leucine, forming the HxxxxxxxxL/V motif. Additionally, the conserved glutamine or histidine residue in the second motif (DxxxQ/H) of DH domains is usually replaced by valine or leucine in EI domains [60].

The olefin shift in cis-AT PKSs is less studied [64]. Detailed studies have been conducted on three cis-AT PKSs that produce polyketides containing β,γ double bonds: ansamitocin [65], ambruticin [66], and gephyronic acid [67]. Similar stereochemical results have been reported for several other cis-AT PKS-produced polyketides, such as disciformycins A,B [68], chax-amycin [69], chondrocloren [70], crocacin [71], cryptophycin [72], divergolide [73], hygrocin [74], naphtomycin [75], and rifamycin [76]. The sequences of the isomerizing DH domains in cis-AT PKSs do not feature the characteristic substitution of proline for a hydrophobic amino acid typically seen in EI domains.

An alternative mechanism for the formation of the hemiketal ring (Path 2, Figure 5B) implies that all DH domains in the *iru* PKS are active, leading to the formation of a triene precursor. The six-membered ring could then be formed via 6π-electrocyclization, followed by the addition of a water molecule. For this reaction to occur, the 4-5 double bond must adopt cis-configuration. In polyketide biosynthesis, cis double bond formation is typically mediated by syn-elimination of (3*S*)-alcohols, introduced by A-type KR domains [77]. However, the KR domain of the M11 module (Figure S3) is classified as B-type.

Nonetheless, the presence of an inactive AT domain in M12 suggests an iterative nature of elongation by M11, and stuttering DH modules are known to form cis-configured double bonds from 3l-alcohols [77]. Alternatively, isomerization can be achieved by post-PKS tailoring by isomerase, but the *iru* BGC does not appear to encode proteins predicted to have such a function. However, studies of the biosynthesis of relative pyran-containing natural products suggest that such isomerization and transannular electrocyclization could occur spontaneously upon exposure to light [78]. Although the literature example required UV-A light, the Iru precursor-extended π-system should easily absorb a photon from a visible spectrum for *E*/*Z* isomerization. While electrocyclization into a pyran ring is usually an endothermic process [79,80], incorporation into a macrocycle can make it more energetically favorable.

**Figure 6:**
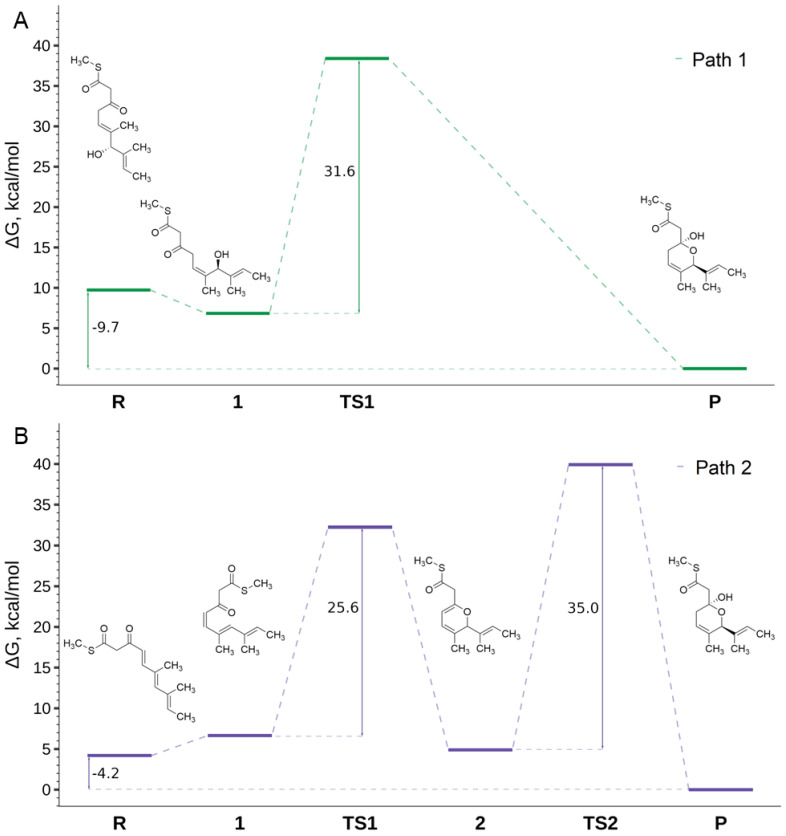
Free energy profiles for Path 1 (**A**) and Path 2 (**B**). Energy differences are given in kcal/mol.

We examined the activation energies for both potential reaction pathways to assess the feasibility of a spontaneous transformation of two distinct reactants into the desired product under physiological conditions. The corresponding Gibbs free energy changes are shown in Figure 6. Typically, cis–trans isomerization of a carbon–carbon double bond proceeds via the formation of a biradical intermediate, which precludes proper treatment under a one-electron formalism and was therefore excluded from this study. It is worth noting that the yield of spontaneous alkene cis–trans isomerization in vivo is negligible, and this reaction is generally enzyme-catalyzed.

For both reaction pathways, the ΔG_1–R_ energy is close to zero, measuring −2.9 kcal/mol for Path 1 and 2.45 kcal/mol for Path 2, suggesting that the reaction could proceed from a thermodynamic point of view. The subsequent cyclization reaction is more favorable in Path 2 (TS1 = 25.6 kcal/mol) than in Path 1 (31.6 kcal/mol). Notably, the cyclization reaction in Path 1 requires an external water molecule, which plays a critical role in proton transfer (see TS1 geometries in Supplementary Materials) and ultimately leads to product formation. In both pathways, the cyclization reaction is exothermic, with ΔG_P–1_ = −6.84 kcal/mol in Path 1 and ΔG_2–1_ = −1.76 kcal/mol in Path 2.

In Path 2, the formation of the final product from intermediate 2 requires the addition of a water molecule. According to our findings, the activation barrier of this process (TS2) is 35 kcal/mol, requiring a protonated carboxyl group as a proton donor and an external water molecule to provide the hydroxyl. During this reaction, both a proton and a hydroxyl group are attached to intermediate 2, while the remaining hydrogen from the water molecule reprotonates the carboxyl group (see TS2 geometry in Supplementary Materials). In living organisms, this reaction is typically catalyzed by hydrolases and would not proceed without enzymatic assistance; however, in the present case, this process could occur, albeit at a very low rate.

Finally, both pathways are overall exothermic, with ΔG_P–R_ = −9.73 kcal/mol for Path 1 and ΔG_P–R_ = −4.2 kcal/mol for Path 2, indicating that product formation is thermodynamically favorable. In line with these results, the final product could theoretically be obtained through either reaction pathway, although the process is significantly limited by kinetic barriers.

**Figure 7:**
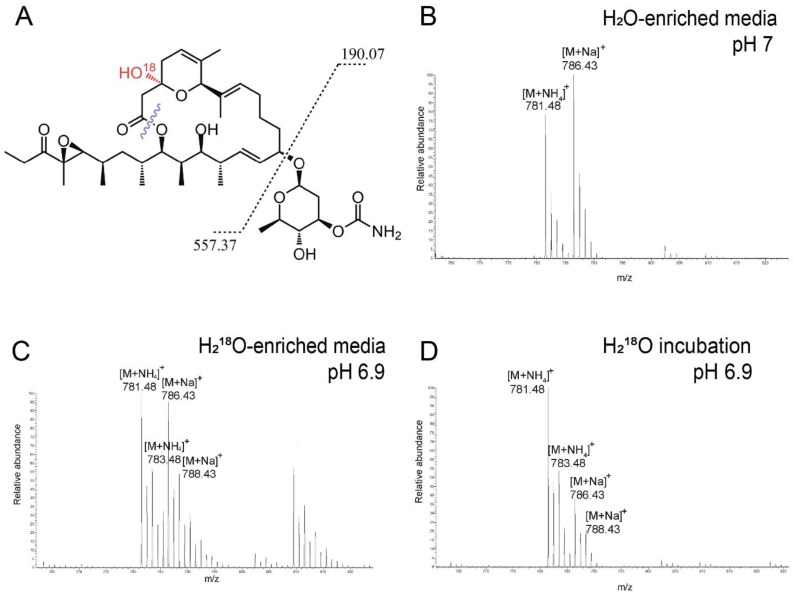
(**A**) MS/MS fragmentation pathway of ^18^O-labeled Iru. Positive-mode mass spectrum of Iru from unlabeled media (**B**), H_2_^18^O-enriched media (**C**). Positive-mode mass spectrum of a standard of Iru incubated in H_2_^18^O for 24 h at pH 6.9 (**D**).

To evaluate the spontaneous water addition during the biosynthesis of Iru, the producing strain was cultivated in media containing 50% H_2_^18^O. We observed the incorporation of the ^18^O isotope in the mass spectra of Iru grown in ^18^O-enriched media (Figure 7C). This was evidenced by the appearance of the [M+NH_4_]⁺ and [M+Na]⁺ adducts at *m*/*z* 783.48 and 788.43, respectively. We incubated the Iru standard in ^18^O-enriched media for 24 h at the same pH as the growth media. After incubation, we observed the same mass spectral pattern as for Iru grown in ^18^O-enriched media, indicating that ^18^O incorporation occurs at this pH both during biosynthesis and under these incubation conditions (Figure 7C,D). These data suggest that the hydroxyl group in the hemiketal ring is readily exchangeable. This result is consistent with previous reports of an O-methylated venturicidin analog, which was presumably formed via exchange with methanol at this position during the isolation process [38].

## 3. Materials and Methods

### 3.1. Genome Sequencing

Genomic DNA was isolated from *Streptomyces kanamyceticus* INA-Ac-5812 using the PowerSoil DNA isolation kit (Mo Bio Laboratories Inc., Carlsbad, CA, USA). The sequencing library for Illumina sequencing was prepared using the NEBNext Ultra II DNA Library Prep Kit (New England Biolabs, Ipswich, MA, USA) following the manufacturer’s instructions. The sequencing of this library on the Illumina HiSeq-2500 platform using HiSeq Rapid Run v2 sequencing reagents generated 32,609,767 single-end reads with an average length of 250 nt (8.2 Gbp in total). Primer sequences were removed from the Illumina reads using Cutadapt v.1.17 [81] with the default settings, and low-quality read ends were trimmed using Sickle v.1.33 (option q = 30) (https://github.com/najoshi/sickle, accessed on 20 August 2019). For Nanopore sequencing, the library was prepared using a 1D ligation sequencing kit (SQK-LSK108, Oxford Nanopore, Oxford, UK). Sequencing of this library in an R9.4 flow cell (FLO-MIN106) using a MinION device yielded 158026 reads with a total length of 1.1 Gbp. Hybrid assembly of Illumina and Nanopore reads was performed using Unicycler ver. 0.4.8 [82]. A complete linear genome of 8,957,691 bp was obtained. Gene search and annotation were performed using the RAST server [83], followed by manual correction. The genome of *Streptomyces kanamyceticus* INA-Ac-5812 was analyzed with antiSMASH [84] to identify potential biosynthetic gene clusters. Homologous gene clusters were identified with MultiGeneBlast [85] using the MIBiG database [86]. Putative functions of tailoring enzymes were assigned according to the predicted function of the closest characterized relative identified by Blast search [87] in NCBI. The complete genome sequence of *Streptomyces kanamyceticus* INA-Ac-5812 was submitted to the NCBI GenBank database and is accessible under the number CP172446.

### 3.2. Computational Details

Initial geometries for reactants, intermediates, and products were manually created in Avogadro 1.2 software [88] and pre-optimized using the MMFF94s force field [89]. Conformational analysis was performed for all mentioned geometries using the CREST algorithm, ver. 2.12 [90] and the xTB package [91], ver. 6.4.0. The GFN1-xTB method [92] with the ALPB water model [93] was employed. The optimal geometries were subsequently used for DFT optimization. DFT optimization was performed in ORCA 5.0.3 software [94]. All geometries were optimized using a composite GGA B97-3c functional [95] with a built-in triple-ζ basis set. The DIIS (direct inversion in iterative subspace) procedure was applied [96]. RI-J Coloumb energy approximation was utilized. Numerical Hessian at 298.15 K was calculated. Transition state geometries were obtained using the NEB-TS algorithm [97] with subsequent OptTS optimization. Additionally, for all structures, electron energy was updated using ωB97X-V/def2-TZVP [98] single-point calculations. RIJCOSX approximation [99] was utilized. Solvation effects were captured using B3LYP/6-31G* [100] single-point calculations with the SMD solvation model [101]. Single-point gas-phase energy was subtracted from single-point energy with the SMD model, as described in a recent study [102]. Strict convergence criteria for geometry optimization and the SCF were established (10^−6^ and 10^−8^ a.u., respectively), and the SlowConv SCF convergence method was applied. The DEFGRID2 parameter was defined. All calculations were performed using spin-restricted Kohn–Sham formalism. The Fock matrix was updated on each step of the SCF calculation. Second-order SCF approximation was turned off (NOSOSCF parameter) [103]. Structures were rendered using PovRay software, ver. 3.7.0.10.

### 3.3. LC–MS

LC–MS analysis was carried out on an Ultimate 3000 RSLC HPLC system connected to a QExactive Plus mass spectrometer (Thermo Fisher Scientific, Waltham, MA, USA). Samples were separated on a Gemini C18 3 µm NX LC column 100 × 2.1 mm (Phenomenex) at 200 μL/min flow rate. Separation was done by a gradient of 90% acetonitrile (LiChrosolv, LCMS grade) in water (LiChrosolv, LCMS grade), 10 mM ammonium formate (Sharlab, LCMS grade), 0.1% FA (LiChrosolv, LCMS grade) (Buffer B) in 99.9% H_2_O, 10 mM ammonium formate, 0.1% FA (Buffer A). UV data was collected at 220 nm. MS1 and MS2 spectra were recorded at 30 K and 15 K resolution respectively with HCD fragmentation.

### 3.4. Cultivation and Chemical Supplementation

Iru isolation and structure elucidation were reported previously [28]. Cultivation of the Iru-producing strain *Streptomyces kanamyceticus* INA-Ac-5812 with the addition of heavy water H_2_^18^O (50%) was carried out in 1 mL of ISP2 medium. Composition of ISP2 medium (g/L): Glucose 4, Yeast extract 4, Malt extract 10. The inoculum was grown on the ISP2 medium in a shaker incubator at 120 rpm for 7 days at 28 °C. The heavy water experiment was conducted in two variants: pH-controlled (with phosphate buffer) and standard cultivation.

Glucose was dissolved in 2 mL of H_2_O/PBS 7.4 (autoclaved separately), and 1 mL H_2_^18^O (50%) and 1 mL H_2_O were sterilized separately. Yeast extract and malt extract were dissolved in 3 mL of H_2_O/PBS 7.4. After sterilization, 2 mL of glucose and 3 mL of H_2_O/PBS 7.4 were added. In 1 well of a 24-well plate, 500 µL of medium in H_2_O/PBS 7.4 and 500 µL of H_2_^18^O (50%) were combined. In the control well, 500 µL of medium in H_2_O/PBS 7.4 and 500 µL of sterile water were mixed. The pH of the medium in both wells was controlled, and 100 µL of inoculum were added to each well. The plate was incubated in a shaker incubator at 120 rpm for 7 days at 28 °C.

After incubation, the contents of the wells were transferred to 2 mL tubes centrifuged at 15,000 rcf for 10 min. The supernatant was discarded, and 1 mL of methanol was added to the debris. The biomass in methanol was treated with ultrasound and then centrifuged at 15,000 rcf for 10 min. The methanol extract was evaporated at 45 °C to dryness. Next, 500 μL MeOH (Panreac, HPLC grade) was added to the dried sample, centrifuged at 18,000 rcf for 5 min, and insoluble precipitate was discarded. The supernatant was filtered with a 0.22 μm nylon syringe filter and analyzed by LC–MS.

## 4. Conclusions

This study provides valuable insights into the biosynthetic pathway of Iru, a structurally complex polyketide produced by *Streptomyces kanamyceticus* INA-Ac-5812. By analyzing the biosynthetic gene cluster and investigating the key PKS domains, we proposed mechanisms for the formation of the unique hemiketal ring and cis-double bond in Iru. Our analysis of the unusual module architecture and spatial structure of IruF, along with highly calculated activation energies for the electrocyclization-based pathway, suggest that the olefin shift enabling hemiketal formation is likely to be mediated by modules M11 and M12 of this protein. Additionally, the predicted functions of tailoring enzymes and the comparison with previously described BGCs of related compounds revealed plausible involvement of Iru3 and Iru4 in the epoxidation of Iru. These findings enhance our understanding of the intricate biosynthetic processes that govern polyketide formation and open new avenues for the development of novel analogs with improved biological activities through synthetic biology and biosynthetic engineering.

## Data Availability

The complete genome sequence of *Streptomyces kanamyceticus* INA-Ac-5812 was submitted to the NCBI GenBank database and is accessible under the number CP172446.

## Supplementary Materials

The following supporting information can be downloaded at: https://www.mdpi.com/article/10.3390/antibiotics13121167/s1, Figure S1: KS domain analysis; Figure S2: AT domain analysis; Figure S3: KR domain analysis; Figure S4: structures of irumamnolides I, II; Figure S5. Confidence scores of the IruF/IruE complex; Figure S6. AlphaFold3 model of F420/flavin-dependent oxidoreductase encoded by orf4; Figure S7. AlphaFold3 model of protein of unknown function (DUF5995) encoded by orf3; Table S1: Annotation of iru BGC; Supplemental archive: additional computational details.
