# Supplementary material for: Molecular Decoration and Unconventional Double Bond Migration in Irumamycin Biosynthesis
Source: Antibiotics (Basel). 2024 Dec 3;13(12):1167. doi: 10.3390/antibiotics13121167 (PMC11672594; doi:10.3390/antibiotics13121167)

Molecular Decoration and Unconventional Double Bond Migration in Irumamycin Biosynthesis

Vera A. Alferova [1,*], Anna A. Baranova [1], Olga A. Belozerova [1], Evgeny L. Gulyak [1], Andrey A. Mikhaylov [1], Yaroslav V. Solovev [1], Mikhail Y. Zhitlov [1,2], Arseniy A. Sinichich [1,2], Anton P. Tyurin [1], Ekaterina A. Trusova [1], Alexey V. Beletsky [3], Andrey V. Mardanov [3], Nikolai V. Ravin [3], Olda A. Lapchinskaya [4], Vladimir A. Korshun [1], Alexander G. Gabibov [1] and Stanislav S. Terekhov [1,*]

[1] Shemyakin-Ovchinnikov Institute of Bioorganic Chemistry, Miklukho-Maklaya 16/10, Moscow 117997, Russia; anjabaranowa@list.ru (A.A.B.); o.belozyorova@gmail.com (O.A.B.); mikhaylov_andrey@yahoo.com (A.A.M.); yaroslavsolovev78@gmail.com (Y.V.S.); droplbox38@gmail.com (M.Y.Z.); asinichich@yandex.ru (A.A.S.); anton2rin@gmail.com (A.P.T.); katatrusova532@gmail.com (E.A.T.); v-korshun@yandex.ru (V.A.K.); gabibov@gmail.com (A.G.G.)

[2] Department of Chemistry, Lomonosov Moscow State University, Leninskie Gory 1, Moscow 119991, Russia

[3] Institute of Bioengineering, Research Center of Biotechnology of the Russian Academy of Sciences, Leninsky Prospect 33-2, Moscow 119071, Russia; mortu@yandex.ru (A.V.B.); mardanov@biengi.ac.ru (A.V.M.)

[4] Gause Institute of New Antibiotics, B. Pirogovskaya, 11, Moscow 119021, Russia; lapchinskaya.olda@mail.ru

* Correspondence: alferovava@gmail.com (V.A.A.); sterekhoff@gmail.com (S.S.T.)

Supplementary Information

Contents

Table S1. Annotation of *iru* BGC 3

Figure S1. KS domain analysis 7

Figure S2. AT domain analysis 8

Figure S3. KR domain analysis 9

Figure S4. Structures of irumamanolides I, II 10

Figure S5. Confidence scores of the IruF/IruE complex 11

Figure S6. AlphaFold3 model of F420/flavin-dependent oxidoreductase encoded by orf4 12

Figure S7. AlphaFold3 model of protein of unknown function (DUF5995) encoded by orf3 13

Table S1. Annotation of *iru* BGC

Strand	*Gene name*	Gene size (bp)	Proposed function	Protein [Organism]. Corresponding to Gene with Sequence Similarity	NCBI Gene Bank Accession Number	ven BGC homologue (BGC0002454)	Indentity % (Cover %)
+	*iru1*	1401		nitrate reductase [Streptomyces sp. WAC 01529]	WP_125511533.1		
+	*iru2*	1203		MFS transporter [Streptomyces formicae]	WP_098241255.1		
+	*iru3*	705		hypothetical protein [Streptomyces huasconensis]	WP_127908983.1		
+	*iru4*	930		LLM class F420-dependent oxidoreductase [Streptomyces formicae]	WP_098241257.1		
-	*iru5*	1692		NAD(P)/FAD-dependent oxidoreductase [Streptomyces huasconensis]	WP_127908982.1		
-	*iru6*	798		carbon-nitrogen hydrolase family protein [Streptomyces alboniger]	WP_055526690.1		
+	*iru7*	630		hypothetical protein [Streptomyces alboniger]	WP_055526610.1		
-	*iru8*	456	Regulation	MULTISPECIES: Lrp/AsnC family transcriptional regulator [Streptomyces]	WP_030787310.1		

-	*iru9*	1464	Transport	MULTISPECIES: amino acid permease [Streptomyces]	WP_055699658.1		
-	*iru10*	1386		GuaB1 family IMP dehydrogenase-related protein [Streptomyces sp. WAC 01529]	WP_125511538.1		
+	*iru11*	879	Sugar biosynthesis	MULTISPECIES: glucose-1-phosphate thymidylyltransferase RfbA [Streptomyces]	WP_055699655.1	*venA*	76 (91)
+	*iru12*	975	Sugar biosynthesis	MULTISPECIES: dTDP-glucose 4,6-dehydratase [Streptomyces]	WP_055699654.1	*venB*	80 (100)
-	*iru13*	1542	Transport	ABC transporter ATP-binding protein [Streptomyces luteocolor]	WP_069883448.1	*venC*	69 (99)
-	*iru14*	1725	Transport	ABC transporter ATP-binding protein [Streptomyces sparsogenes]	WP_065967312.1	*venD*	75 (92)
-	*iru15*	1794	Sugar biosynthesis	MULTISPECIES: carbamoyltransferase [Streptomyces]	WP_031100308.1	*venE*	92 (100)
-	*iruF*	9981	Scaffold biosynthesis, T1PKS (M11, M12)	acyl transferase domain-containing protein [Streptomyces argenteolus]	TWF48114.1	*venF*	85 (100)
-	*iruE*	10935	Scaffold biosynthesis, T1PKS (M10, M9)	type I polyketide synthase [Streptomyces sparsogenes]	WP_076971296.1	*venG*	87 (100)
-	*iruD*	6558	Scaffold biosynthesis, T1PKS (M8)	type I polyketide synthase [Streptomyces luteocolor]	WP_069883583.1	*venH*	90 (100)

-	*iruC*	15714	Scaffold biosynthesis, T1PKS (M5,M6,M7)	type I polyketide synthase [Streptomyces silaceus]	WP_055697323.1	*venI*	86 (98)
-	*iruB*	11280	Scaffold biosynthesis, T1PKS (M3,M4)	type I polyketide synthase [Streptomyces sparsogenes]	WP_076971294.1	*venJ*	85 (100)
-	*iruA*	10938	Scaffold biosynthesis, T1PKS (LM)	type I polyketide synthase [Streptomyces silaceus]	WP_079036345.1	*venK*	78 (100)
+	*iru22*	1500	Sugar biosynthesis	MULTISPECIES: NDP-hexose 2,3-dehydratase [Streptomyces]	WP_107101853.1	*venL*	77 (95)
+	*iru23*	1035	Sugar biosynthesis	Gfo/Idh/MocA family oxidoreductase [Streptomyces sp. NRRL S-4]	WP_078958809.1	*venM*	80 (98)
+	*iru24*	738	Sugar biosynthesis	NAD-dependent epimerase/dehydratase family protein [Streptomyces sp. NRRL F-5650]	WP_031046946.1	*venN*	77 (100)
+	*iru25*	1254	Glycosyltransferase	MULTISPECIES: glycosyltransferase [Streptomyces]	WP_055697328.1	*venO*	87 (100)
+	*iru26*	747	Thioesterase	thioesterase [Streptomyces silaceus]	WP_055697329.1	*venP*	74 (99)
-	*iru27*	372		barstar family protein [Streptomyces huasconensis]	WP_127908978.1		
-	*iru28*	1029	Regulation	transcriptional regulator [Streptomyces sp. NRRL S-920]	WP_037897561.1		

-	*iru29*	681	Sugar biosynthesis	ribulose-phosphate 3-epimerase [Streptomyces huasconensis]	WP_127908976.1		
+	*iru30*	759		extensin-like [Ixodes scapularis]	XP_029832010.1		
-	*iru31*	1461		rRNA cytosine-C5-methyltransferase [Streptomyces sp. WAC 01529]	WP_125511541.1		
-	*iru32*	945		methionyl-tRNA formyltransferase [Streptomyces huasconensis]	WP_127908975.1		
+	*iru33*	561		MULTISPECIES: hypothetical protein [Streptomyces]	WP_055697336.1		
-	*iru34*	2181		Select seq ref|WP_030787277.1| primosomal protein N' [Streptomyces sp. NRRL S-920]	WP_030787277.1		
-	*iru35*	1209		methionine adenosyltransferase [Streptomyces atriruber]	WP_055564207.1		
-	*iru36*	1245		bifunctional phosphopantothenoylcysteine decarboxylase/phosphopantothenate--cysteine ligase CoaBC [Streptomyces venezuelae]	WP_150182811.1		

Figure S1. KS domain analysis

LM KS	D	S	G	**Q**	S	S	S
M1 KS	D	T	A	**C**	S	S	S
M2 KS	D	T	A	**C**	S	S	S
M3 KS	D	T	A	**C**	S	S	S
M4 KS	D	T	A	**C**	S	S	S
M5 KS	D	T	A	**C**	S	S	S
M6 KS	D	T	A	**C**	S	S	S
M7 KS	D	T	A	**C**	S	S	S
M8 KS	D	T	A	**C**	S	S	S
M9 KS	D	T	A	**C**	S	S	S
M10 KS	D	T	A	**C**	S	S	S
M11 KS	D	T	A	**C**	S	A	S
M12 KS	D	T	A	**C**	S	S	S
	D	T	X	C	S	X	S

Figure S2. AT domain analysis

LM	R	V	D	V	V	Q	G	H	S	Q	G	E	Y	G	S	H
M1	K	V	D	V	L	Q	G	H	S	Q	G	E	F	A	S	H
M2	R	V	D	V	V	Q	G	H	S	Q	G	E	Y	G	S	H
M3	R	V	D	V	V	Q	G	H	S	Q	G	E	Y	G	S	H
M4	R	V	D	V	I	Q	G	H	S	Q	G	E	Y	G	S	H
M5	R	V	D	V	V	Q	G	H	S	Q	G	E	Y	A	S	H
M9	R	V	D	V	V	Q	G	H	S	Q	G	E	Y	A	S	H
M10	R	V	D	V	V	Q	G	H	S	Q	G	E	Y	P	S	H
Motif	X	**V**	**D**	**V**	X	**Q**	**G**	**H**	**S**	**Q**	**G**	**E**	X	X	**S**	**H**
M6	E	T	V	Y	T	Q	G	H	S	V	G	E	Q	A	F	H
M7	E	T	V	Y	T	Q	G	H	S	V	G	E	Q	A	F	H
M11	R	T	G	Y	T	Q	G	H	S	I	G	E	H	A	F	H
M8	R	D	D	V	V	Q	G	H	S	Q	G	E	Y	A	S	H
Motif	X	**T**	X	**Y**	**T**	**Q**	**G**	**H**	**S**	**V/I**	**G**	**E**	X	**A**	**F**	**H**
M12	D	P	A	V	A	H	G	I	G	G	G	E	V	T	A	R

Figure S3. KR domain analysis

KR M2	H T A A V **L D D** G A L	S S L A G T V G M A G Q G N Y A **P** G N A
KR M3	H A A G A **L D D** G V V	S S A A G A L G S A G Q G G Y A A A N S
KR M4	H T A A A **L D D** G P L	S S T A G T F G A A G Q G N Y A **P** G N A
KR M5	H A A G V **L D D** G V L	S S A A G V F G A P G Q G N Y A A A N A
KR M6	H S A G V V D D G V I	S S A A G I L G A V G **Q** A N Y A A A N T
KR M7	H T A G V A Q S T R I	S S T A G V **W** G G A G **Q** G A Y G A A N A
KR M8	H A A G V I D D G V L	S S A S G V F G A P G **Q** A N Y A A A N A
KR M9	H A A G A V D D G V V	S S A A G V F G S P G **Q** A N Y A A A N V
KR M10	H A A G A V D D G V V	S S A A G V F G S P G **Q** A N Y A A A N V
KR M11	H A A G V **L D D** G M V	S S A A G V F G S P G Q S N Y A A A N T

Figure S4. Structures of irumamanolides I, II

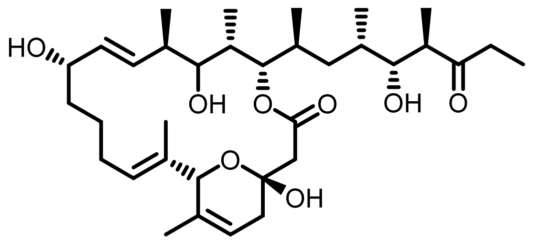

Irumamanolide II

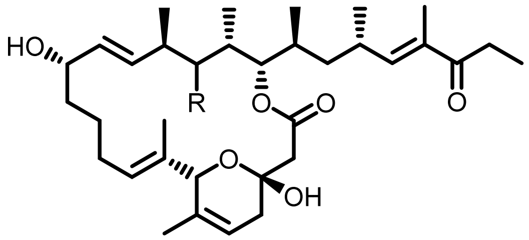

Irumamanolide I

Figure S5. Confidence scores of the IruF/IruE complex

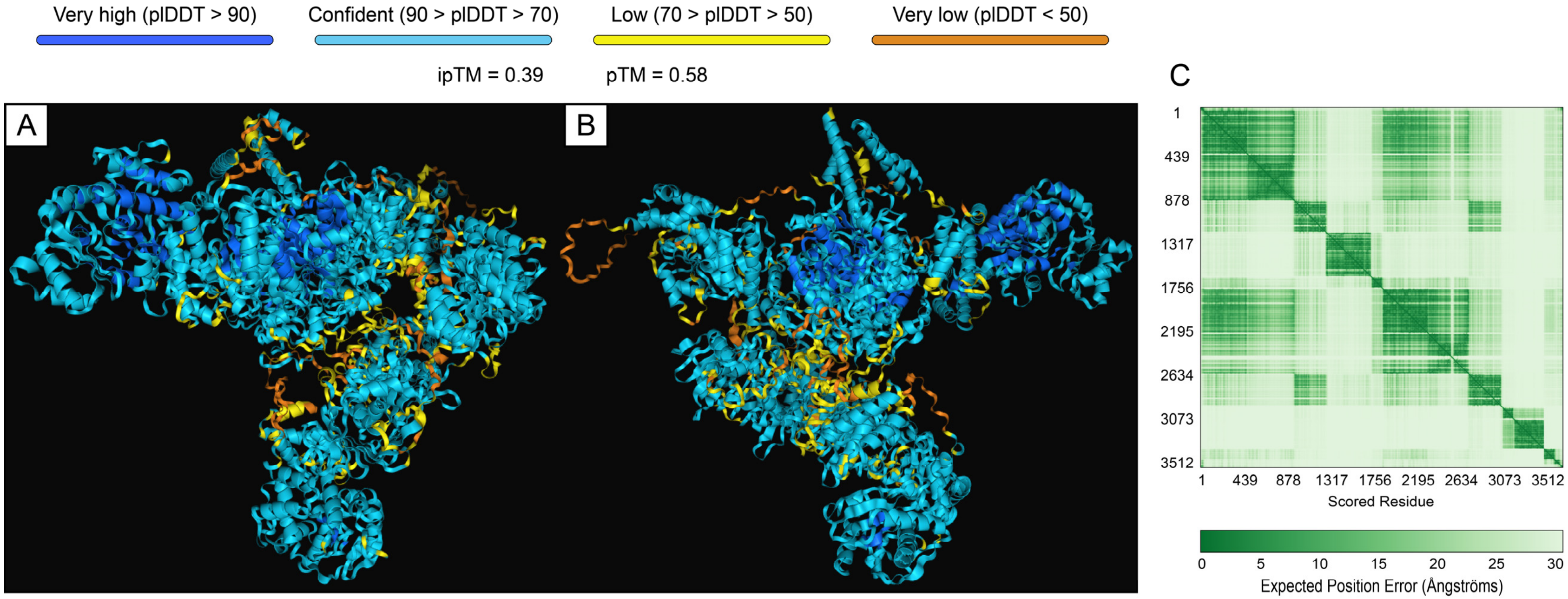

Figure S5. Confidence scores of the IruF/IruE complex from Figure 3 projections (A) and (B). Expected Position Errors are indicated (C).

Figure S6. AlphaFold3 model of F420/flavin-dependent oxidoreductase encoded by orf4

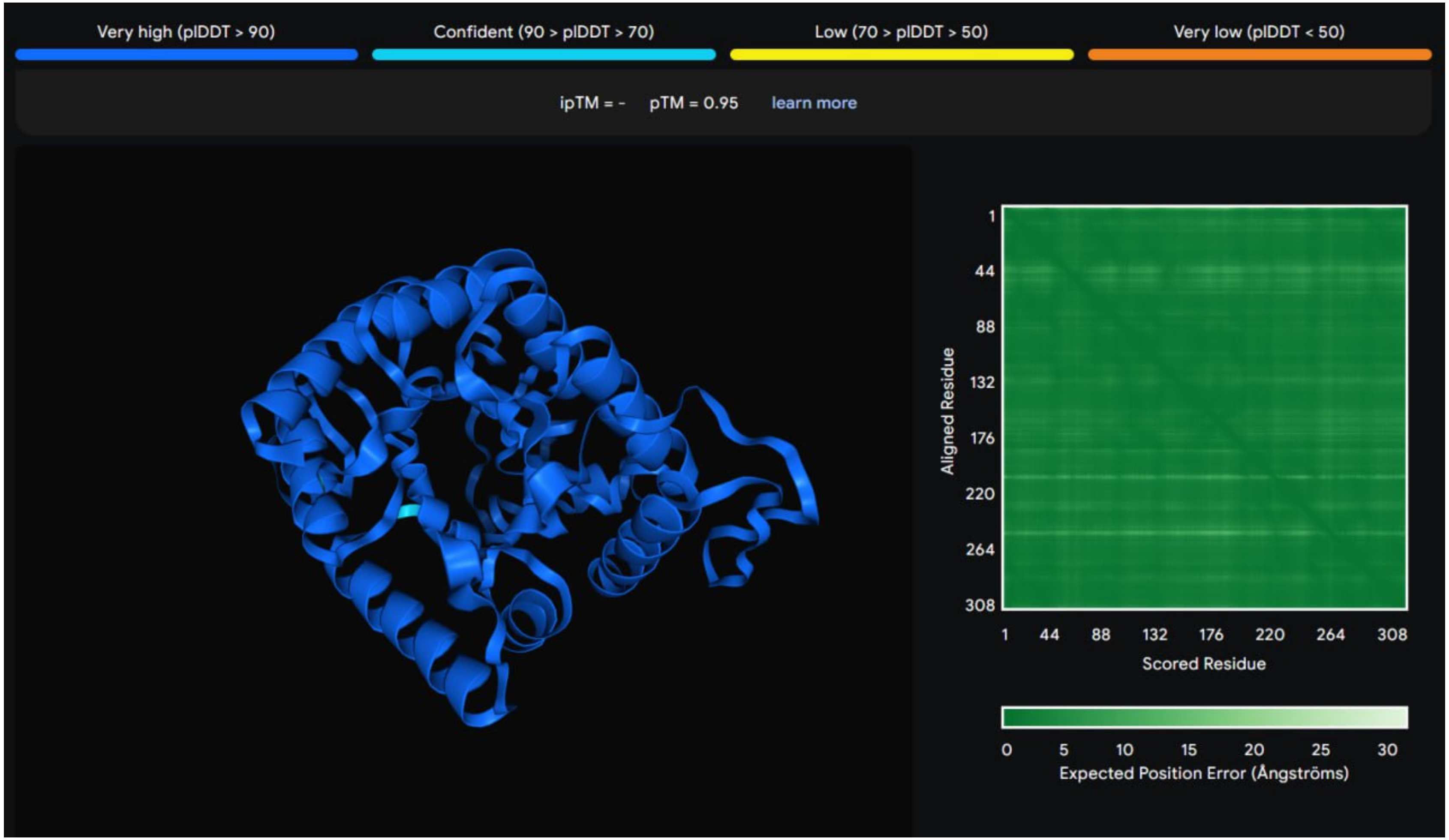

Figure S7. AlphaFold3 model of protein of unknown function (DUF5995) encoded by orf3

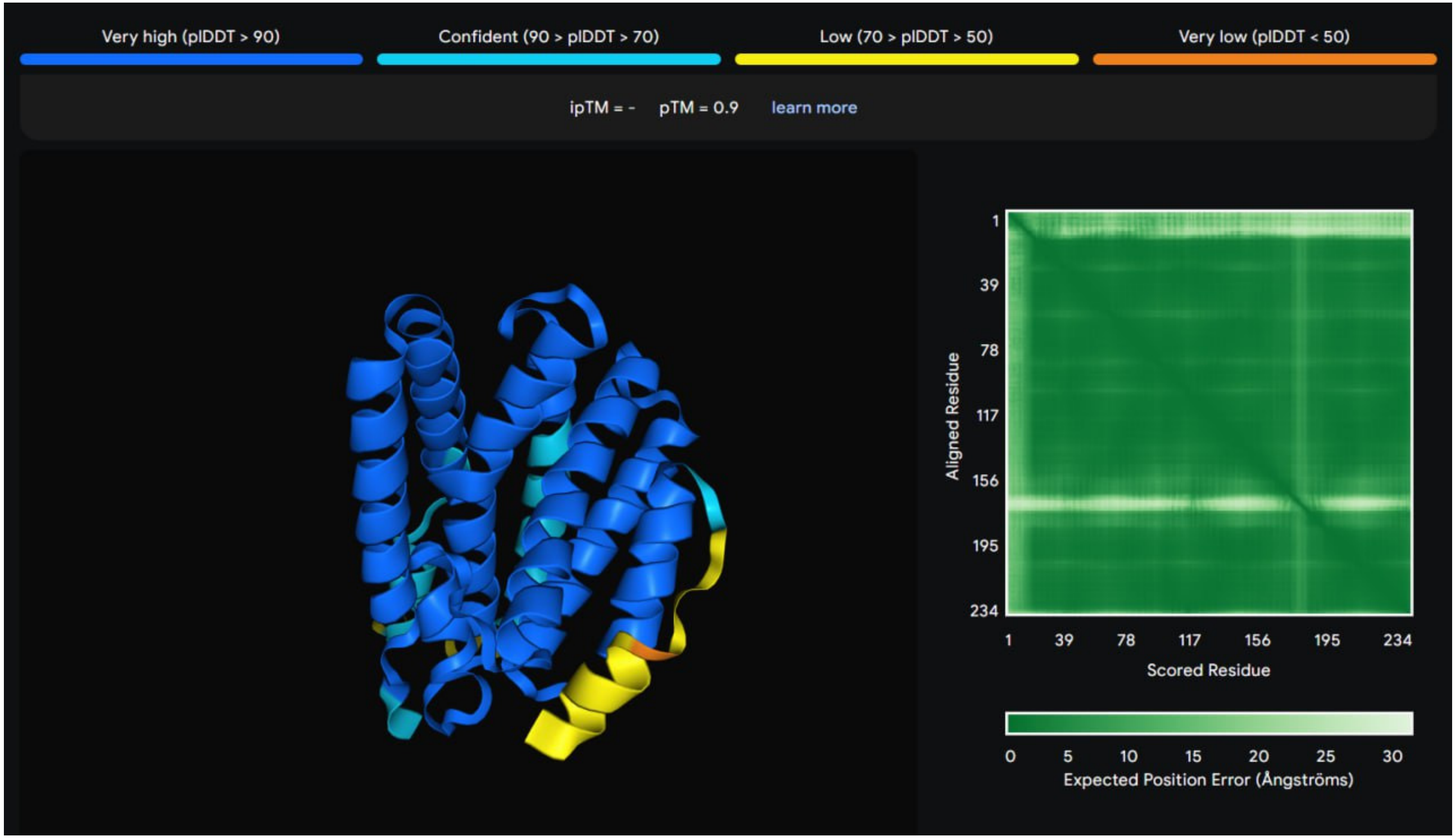

Supplement: Supplementary file 1 [file antibiotics-13-01167-s001.zip › antibiotics-3329364-supplementary.pdf]
